# Pyruvate kinase M2 sustains cardiac mitochondrial integrity in septic cardiomyopathy by regulating PHB2-dependent mitochondrial biogenesis

**DOI:** 10.7150/ijms.94577

**Published:** 2024-04-08

**Authors:** Jiaxi Ren, Bin Ren, Tong Fu, Yanchun Ma, Ying Tan, Shuxiang Zhang, Yan Li, Qi Wang, Xing Chang, Ying Tong

**Affiliations:** 1 Luoyang Branch of Dongzhimen Hospital Affiliated to Beijing University of Chinese Medicine, Luoyang Hospital of TCM, Luoyang 471000, China.; 2 Daqing Oilfield General Hospital, Daqing 163000, China.; 3 Brandeis University, Waltham, MA 02453, USA.; 4 Heilongjiang University of Chinese Medicine, Harbin 150040, China.; 5 First Afliated Hospital, Heilongjiang University of Chinese Medicine, Harbin 150040, China.; 6 Guang'anmen Hospital, China Academy of Chinese Medical Sciences, Beijing, 100053, China.

**Keywords:** PKM2, PHB2, septic cardiomyopathy, mitochondrial biogenesis

## Abstract

Previous studies have highlighted the protective effects of pyruvate kinase M2 (PKM2) overexpression in septic cardiomyopathy. In our study, we utilized cardiomyocyte-specific *PKM2* knockout mice to further investigate the role of PKM2 in attenuating LPS-induced myocardial dysfunction, focusing on mitochondrial biogenesis and prohibitin 2 (PHB2). Our findings confirmed that the deletion of *PKM2* in cardiomyocytes significantly exacerbated LPS-induced myocardial dysfunction, as evidenced by impaired contractile function and relaxation. Additionally, the deletion of *PKM2* intensified LPS-induced myocardial inflammation. At the molecular level, LPS triggered mitochondrial dysfunction, characterized by reduced ATP production, compromised mitochondrial respiratory complex I/III activities, and increased ROS production. Intriguingly, the absence of PKM2 further worsened LPS-induced mitochondrial damage. Our molecular investigations revealed that LPS disrupted mitochondrial biogenesis in cardiomyocytes, a disruption that was exacerbated by the absence of PKM2. Given that PHB2 is known as a downstream effector of PKM2, we employed PHB2 adenovirus to restore PHB2 levels. The overexpression of PHB2 normalized mitochondrial biogenesis, restored mitochondrial integrity, and promoted mitochondrial function. Overall, our results underscore the critical role of PKM2 in regulating the progression of septic cardiomyopathy. PKM2 deficiency impeded mitochondrial biogenesis, leading to compromised mitochondrial integrity, increased myocardial inflammation, and impaired cardiac function. The overexpression of PHB2 mitigated the deleterious effects of *PKM2* deletion. This discovery offers a novel insight into the molecular mechanisms underlying septic cardiomyopathy and suggests potential therapeutic targets for intervention.

## Introduction

Sepsis-induced cardiomyopathy is a condition characterized by reversible cardiac dysfunction that manifests during severe sepsis or septic shock 1. Recent advancements in research have significantly enhanced our understanding of the underlying mechanisms and potential therapeutic avenues for this complex disorder. Investigations have revealed that the dysregulation of various molecular pathways, encompassing inflammation, oxidative stress, and metabolic perturbations, plays a pivotal role in the pathogenesis of sepsis-induced cardiomyopathy 2-4. Notably, the interplay between pyruvate kinase M2 (PKM2) and prohibitin 2 (PHB2) has emerged as a critical determinant influencing the development of this condition 5. Therapeutic strategies for sepsis-induced cardiomyopathy are geared towards targeting the fundamental mechanisms driving cardiac dysfunction. These approaches may involve interventions to alleviate inflammation, oxidative stress, and metabolic imbalances, alongside efforts to preserve mitochondrial function and cellular energetics 6, 7. Furthermore, novel treatments that specifically target key molecular pathways, such as the PKM2-PHB2 axis 5, show promise in enhancing outcomes for individuals with sepsis-induced cardiomyopathy. The progressive comprehension of the molecular intricacies underpinning sepsis-induced cardiomyopathy, coupled with the advancement of tailored therapies, offers optimism for more effective management of this critical condition in the coming years.

Pyruvate kinase M2 (PKM2) is a specific isoform of the pyruvate kinase enzyme, crucial for cellular metabolism, particularly in glycolysis. Its involvement spans various physiological and pathological processes, including cell proliferation, metabolic regulation, and tumorigenesis 8-10. The pivotal role of PKM2 in regulating cellular energy metabolism and growth is of profound interest in disease research and therapy. The intricate relationship between PKM2 and mitochondria has garnered attention, with research suggesting that PKM2 interacts with mitochondria through diverse mechanisms, influencing mitochondrial function 11-13. PKM2's ability to translocate to mitochondria directly impacts mitochondrial metabolism and function. Moreover, it indirectly influences mitochondrial function and biogenesis by reshaping cellular metabolism through metabolic reprogramming 14, 15. This interplay is vital for maintaining cellular metabolic equilibrium and is pertinent to understanding the pathogenesis of various diseases, including septic cardiomyopathy. In the context of sepsis-induced cardiomyopathy, PKM2 assumes a significant role in modulating the condition's pathogenesis by regulating cellular metabolism and energy production, crucial for cardiac function maintenance, especially under stress conditions like sepsis 16. Studies suggest that PKM2 may impact sepsis-induced cardiomyopathy through interactions with molecules and pathways, such as prohibitin 2 (PHB2). The PKM2-PHB2 axis is implicated in influencing mitochondrial function, oxidative stress, and inflammation—key factors in sepsis-induced cardiac dysfunction development. By regulating metabolic reprogramming, cellular energetics, and mitochondrial function, PKM2 influences the heart's response to septic insults. Understanding PKM2's specific role in sepsis-induced cardiomyopathy may pave the way for developing targeted therapies to modulate its activity and enhance cardiac outcomes in affected patients.

Prohibitin 2 (PHB2) is a protein primarily localized in the inner mitochondrial membrane, where it plays a pivotal role in preserving mitochondrial structure and function 17. Involved in a myriad of mitochondrial processes such as biogenesis, dynamics, and respiratory chain function, PHB2's relationship with mitochondria is intricate and multifaceted 18-20. Within the mitochondria, PHB2 engages with multiple proteins, forming complexes crucial for maintaining mitochondrial homeostasis. Its regulatory functions extend to mitochondrial morphology, membrane potential, and the assembly of respiratory chain complexes 21-23. Additionally, PHB2 has been identified as a key player in modulating cellular responses to stress, including oxidative stress and apoptosis, through its impact on mitochondrial function 24, 25. Disruptions in PHB2 have been linked to mitochondrial dysfunction and the onset of various diseases, underscoring its significance in preserving mitochondrial health. The interplay between PHB2 and mitochondria is essential for cellular metabolism, energy production, and overall cellular equilibrium 26, 27. A comprehensive understanding of the intricate relationship between PHB2 and mitochondria holds promise for shedding light on the pathogenesis of diverse disorders, such as sepsis-induced cardiomyopathy, and unveiling potential therapeutic targets for intervention.

Mitochondrial biogenesis stands as a pivotal factor in the pathogenesis of sepsis-induced cardiomyopathy 28-30. Amidst sepsis, the heart grapples with substantial metabolic and energetic strains, culminating in mitochondrial impairment and compromised cardiac function. The generation of new mitochondria within cells, known as mitochondrial biogenesis, proves indispensable in upholding cellular energy production and function, especially in organs with high energy demands like the heart 31, 32. In the realm of sepsis-induced cardiomyopathy, fostering mitochondrial biogenesis emerges as a viable therapeutic avenue to bolster cardiac function and enhance patient outcomes 33, 34. By bolstering both the quantity and efficacy of mitochondria, cells can better navigate the metabolic challenges imposed by sepsis and alleviate the adverse impacts on cardiac performance. Research indicates that key players in mitochondrial biogenesis, such as peroxisome proliferator-activated receptor gamma coactivator 1-alpha (PGC-1α) and nuclear respiratory factors, experience dysregulation during sepsis, thereby contributing to mitochondrial dysfunction in the cardiac tissue 35. Strategies aimed at reinstating mitochondrial biogenesis pathways have exhibited promise in preclinical investigations as a protective measure against sepsis-induced cardiac dysfunction 29, 30. Delving into the role of mitochondrial biogenesis in sepsis-induced cardiomyopathy yields valuable insights into the molecular underpinnings of the condition, paving the way for targeted therapies that seek to rejuvenate mitochondrial function and enhance cardiac outcomes for septic patients.

## Methods

### Animal Procurement and Experimental Conditions

C57BL/6 mice obtained from the Model Animal Research Center of Nanjing University, China, were housed in a specific pathogen-free (SPF) facility at the Experimental Animal Center of Sun Yat-sen University in accordance with the institution's animal experimentation guidelines. The mice were maintained in a controlled environment at 20°C with 50% humidity, under a 12-hour light/dark cycle, and provided with food and water ad libitum. Cardiomyocyte-specific *PKM2* knockout mice (*PKM2^Cko^*) were acquired from Cyagen Biosciences, Beijing, China 36. The septic cardiomyopathy model was induced by injecting mice with LPS at a dose of 10mg/kg, followed by a 24-hour monitoring period 37.

### Echocardiography Assessment

Cardiac function was assessed using echocardiography in anesthetized mice. Continuous inhalation of isoflurane (2%) mixed with oxygen was used for anesthesia. Echocardiographic evaluations were performed with a Vevo3100 echocardiography machine featuring an MS-550D 40-MHz frequency probe (VisualSonics). B-mode echocardiography along the long axis was employed to evaluate ventricular function, with the ejection fraction calculated using the formula: %EF = (LV end-diastolic volume - LV end-systolic volume) / (LV end-diastolic volume) × 100%. The echocardiographic data were collected in a blinded manner.

### Extracellular ATP Quantification

Extracellular ATP levels were quantified using the ATP determination kit (Thermo Fisher Scientific, A22066) following the manufacturer's protocol. Each sample was obtained from approximately 1.5-2 x 105 cultured cardiomyocytes in 1 mL of culture medium. To prevent rapid ATP degradation by ATPases like CD39 on the extracellular membrane surface, cardiomyocytes were pre-treated with a CD39 inhibitor, POM1 (20 μM). Following treatment, the cardiomyocyte culture medium was promptly collected by centrifugation and heated at 80°C for 5 minutes to inhibit ATP degradation. Standard or experimental samples were incubated with luciferase and its substrate, D-Luciferin, at 28°C for 15 minutes. The luminescence assay was conducted using a multi-plate reader. Results were determined using a standard curve and normalized based on each sample's protein concentration.

### Adenovirus Generation and Cell Transduction

The adenovirus expressing PHB2 (Ad-PHB2) was constructed using the ViraPower Adenovirus Expression System from Invitrogen following the manufacturer's instructions. As a negative control, adenovirus expressing LacZ (Ad-LacZ) was utilized. Titration of Ad-PHB2 and Ad-LacZ was conducted to determine viral concentrations. Wild-type (WT) mouse cardiomyocytes and *PKM2^Cko^* mouse cardiomyocytes were separately transduced with Ad-PHB2 (50 MOI) or Ad-LacZ (50 MOI), with subsequent removal of the virus after 6 hours. The cardiomyocytes were then cultured for an additional 48 hours before undergoing the specified treatments. Expression levels of PHB2 were assessed via western blot analysis.

### Enzyme-linked Immunosorbent Assay (ELISA)

The activities of endothelial nitric oxide synthase (eNOS), endothelin-1 (ET-1), and mitochondrial respiratory complex I/III were assessed using ELISA kits from R&D Systems, USA, following the provided protocols.

### Cell Culture

Cardiomyocytes were obtained from 8- to 10-week-old C57BL/6N (WT) or *PKM2^Cko^* (KO) mice following established procedures. The primary cardiomyocytes were collected, plated on 6-well plates at a density of 2×10^4^ cells/cm^2^, and cultured in H-DMEM medium. To simulate septic cardiomyopathy* in vitro*, the cardiomyocytes were exposed to 10mg/ml LPS for 6 hours.

### Total RNA Extraction and Quantitative Real-Time Polymerase Chain Reaction (RT-qPCR)

Mice samples were collected, and total RNA was isolated utilizing TRIzol (Invitrogen, Life Technologies, USA) following the manufacturer's guidelines. The quality and concentration of the total RNA were assessed using a UV-visible spectrophotometer (BIOMATE 3 S, Thermo Scientific, USA). Subsequently, the RNA was reverse transcribed into cDNA using the Transcriptor First Strand cDNA Synthesis Kit (Roche Applied Science, USA). The cDNA templates were amplified using a PCR Thermal Cycler (Bio-Rad, USA), and qRT-PCR was conducted with a reverse transcription system (LC-480, Roche, USA) employing SYBR Master Mix (Roche Applied Science). GAPDH served as the housekeeping gene for normalization.

### Immunofluorescence and Confocal Microscopy

Mouse heart tissue samples were fixed in Antigenfix (Diapath, Martinengo, Italy) at 4°C for 1 hour, then rinsed twice in PBS, immersed in 34% sucrose overnight, and embedded in Tissue-Tek OCT compound (Sakura Finetek) before being frozen. Sections of 20 µm thickness were sliced using a cryostat (Leica 1950), rehydrated in PBS for 5 minutes, permeabilized with 0.5% saponin, and blocked for 30 minutes with a mixture of 2% BSA, 1% FBS, and 1% donkey or goat serum. The heart sections were incubated with primary antibodies followed by suitable secondary antibodies. After mounting the slides in ProLong Diamond, imaging was performed using a Zeiss LSM780 confocal microscope (Carl Zeiss, Oberkochen, Germany) equipped with a spectral detector for spectral unmixing. Image analysis was carried out using Imaris software (Oxford Instruments, Zürich, Switzerland).

### Western Blotting Assay

Cell protein extracts were obtained using an ice-cold lysis buffer comprising RIPA buffer and a protease inhibitor cocktail. Nuclear protein fractions were isolated utilizing the Nuclear and Cytoplasmic Extraction Kit (Beyotime Biotechnology, Shanghai, China) as per the provided instructions. The protein concentration was determined using Nano Drop (Thermo Scientific, USA). Subsequently, the proteins were denatured (95°C, 15 min) in 5× SDS loading buffer, and then separated by SDS-polyacrylamide gel electrophoresis. Following transfer onto polyvinylidene difluoride membranes, target proteins were probed with specific primary and secondary antibodies. Protein visualization, blot imaging, and quantification were carried out following established protocols. β-Actin served as the loading control for total proteins, while Lamin B1 was utilized as the loading control for nuclear proteins.

### ROS Measurement

Intracellular ROS levels were assessed using CM-H2DCFDA (Life Technologies, USA). Following the described trypsinization process to release cells from the spheroids, the cells were centrifuged at 1,200 rpm at room temperature for 3 minutes, washed with 1× PBS, and centrifuged again at 1,200 rpm at room temperature for 3 minutes. The fluorescence intensity of CM-H2DCFDA, indicative of intracellular ROS levels, was measured using the NovoCyte Advanteon BVYG analyzer (Agilent, USA).

### Cell Viability Assessment

Cell viability was determined utilizing the Cell Counting Kit-8 (Nanjing Enogene Biotechnology, Nanjing, China). Cells were plated in 96-well plates and exposed to various reagents for 24 hours. Subsequently, 150 μl of CCK-8 solution was added to each well and incubated at 37°C in a 5% CO_2_ environment for 4 hours. The spectrophotometric absorbance at 450 nm was then quantified using the Multi-Detection Microplate Reader (Bio-Rad, Hercules, CA, USA).

### Statistical Analysis

The data are expressed as mean ± SD. Paired or unpaired t-tests were employed, as appropriate, to compare differences between two datasets. For comparisons involving more than two groups, the Kruskal-Wallis test with Dunn's post hoc analysis or One-way Analysis of Variance (ANOVA) followed by Bonferroni multiple comparisons test were conducted based on normality criteria. Statistical analyses were carried out using GraphPad Prism version 5.

## Results

### *PKM2* Deletion Exacerbates LPS-Mediated Cardiac Dysfunction

**Figure 1:**
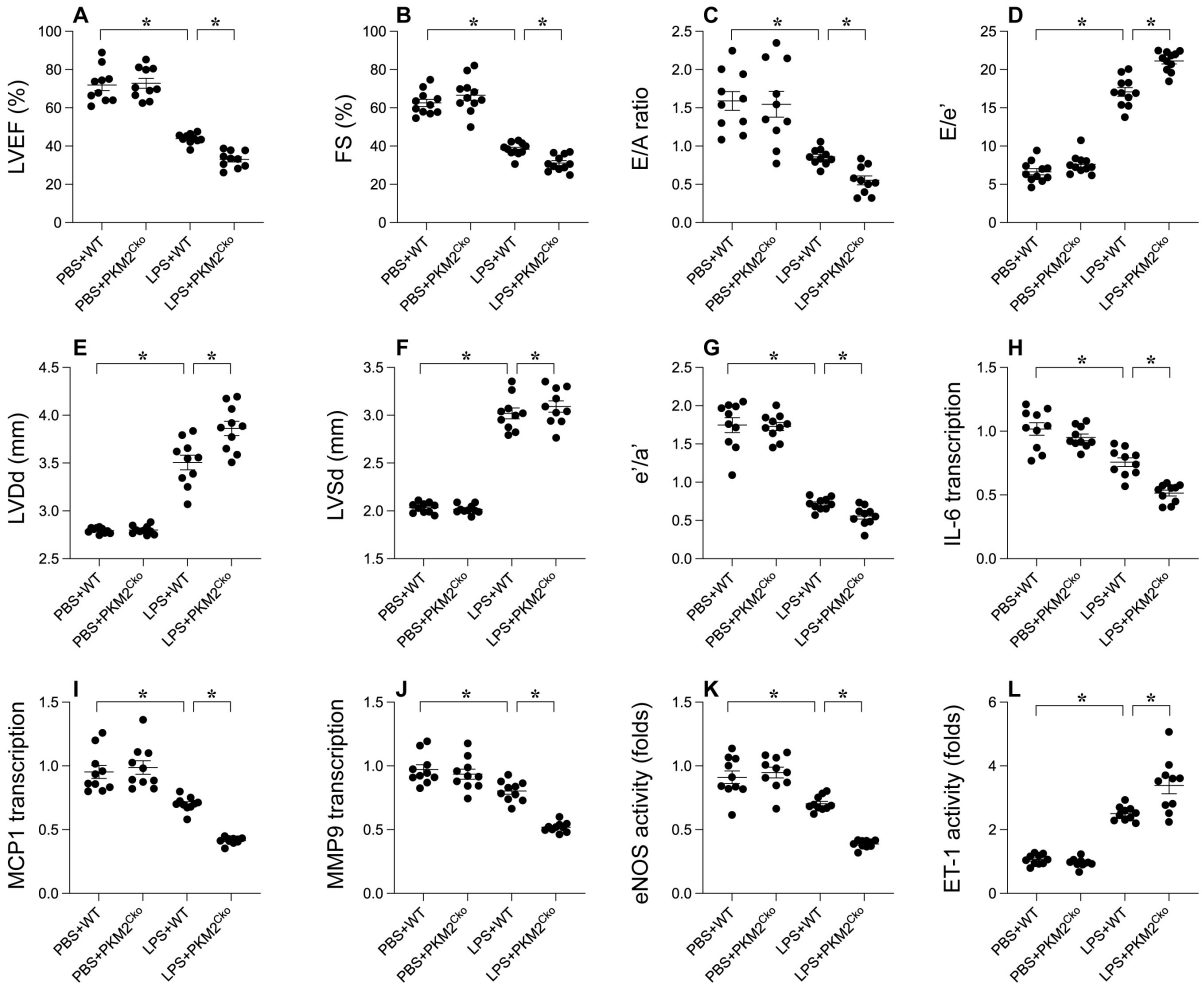
** Impact of *PKM2* Deletion on LPS-Induced Cardiac Dysfunction. A-G.** WT and cardiomyocyte-specific *PKM2* knockout (*PKM2^Cko^*) mice were administered LPS to induce septic cardiomyopathy *in vivo*, with heart function assessed via echocardiography. **H-J.** Inflammation factors like IL-6, MCP1, and MMP9 were analyzed using qPCR. **K-L.** ELISA was utilized to assess eNOS and ET-1 activity. *p<0.05.

To elucidate the role of PKM2 in LPS-induced septic cardiomyopathy, we employed cardiomyocyte-specific *PKM2* knockout mice. Subsequently, these mice were administered LPS to induce septic cardiomyopathy, following which heart function was assessed via echocardiography. In comparison to mice injected with PBS, LPS administration disrupted myocardial contractile parameters and impeded diastolic function (Figure 1A-G). Intriguingly, the absence of PKM2 in the heart exacerbated the impairment in contractile and relaxation functions (Figure 1A-G). Given that the inflammatory response is a key driver in the progression of septic cardiomyopathy, we utilized qPCR and ELISA to evaluate inflammation within the myocardium. The transcription of IL-6, MCP1, and MMP9 exhibited rapid upregulation in response to LPS (Figure 1H-J). Notably, PKM2 deficiency further intensified the transcription levels of IL-6, MCP1, and MMP9 (Figure 1H-J). Additionally, we observed an elevation in ET-1 activity coupled with a reduction in eNOS activity, indicating micro-vessel constriction in the heart under LPS conditions (Figure 1K-L). Notably, the absence of PKM2 amplified ET-1 activity and suppressed eNOS activity in the heart (Figure 1K-L). Collectively, our findings underscore that LPS-induced septic cardiomyopathy is exacerbated by the deletion of *PKM2*.

### *PKM2* Deletion Induces LPS-Induced Cardiomyocyte Oxidative Stress and Apoptosis

**Figure 2:**
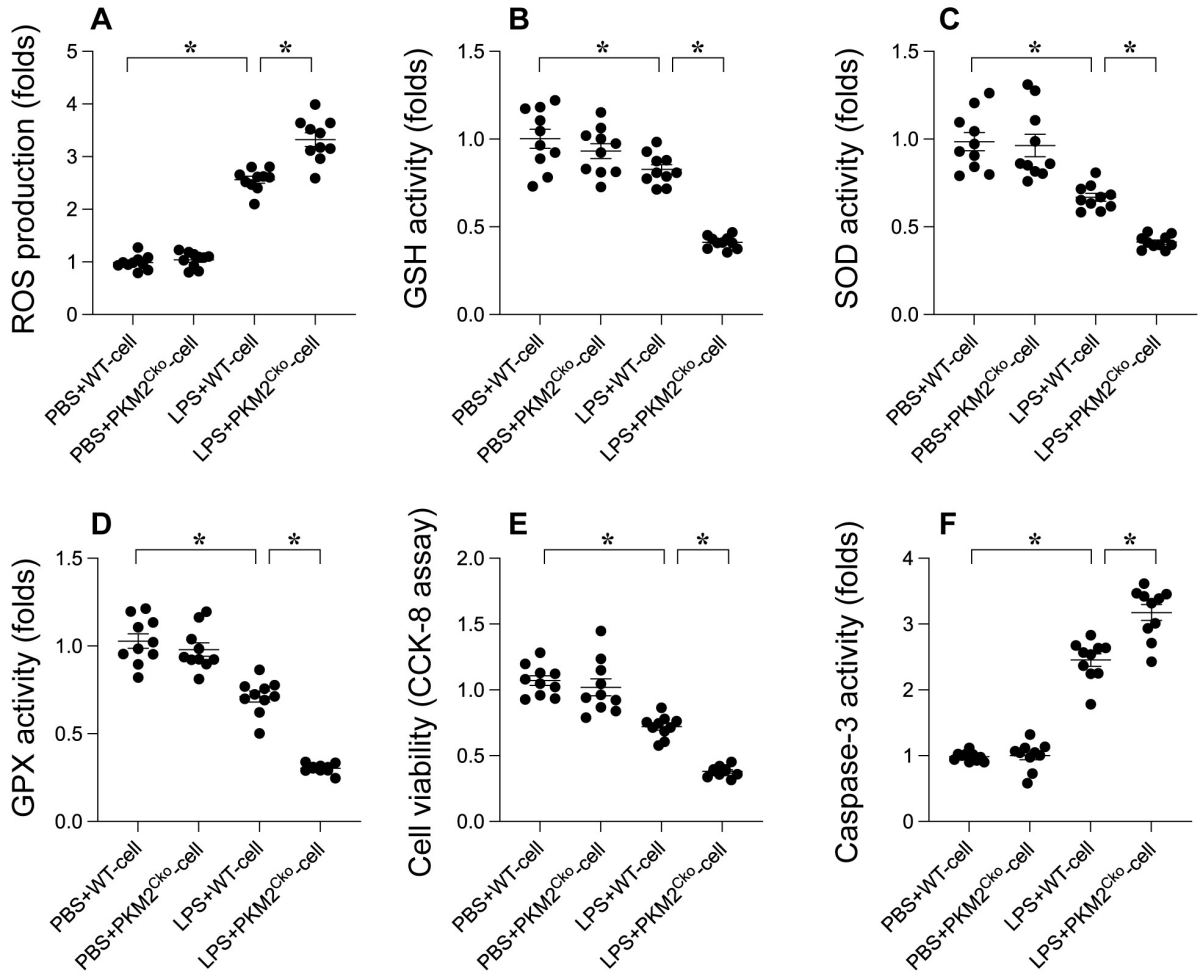
** Role of *PKM2* Deletion in LPS-Induced Cardiomyocyte Oxidative Stress and Apoptosis. Cardiomyocytes from WT and P*KM2^Cko^* mice were treated with LPS *in vitro*. A.** Immunofluorescence detected ROS levels in cardiomyocytes post-LPS exposure.** B-D.** ELISA kits evaluated GSH, GPX, and SOD activities in response to LPS.** E.** Cell viability post-LPS treatment was assessed via CCK-8 assay. **F.** Caspase-3 activity was measured using an ELISA kit. *p<0.05.

Oxidative stress and apoptosis are recognized as key molecular mechanisms contributing to septic cardiomyopathy. We investigated whether PKM2 deficiency exacerbates the progression of septic cardiomyopathy by enhancing oxidative damage and promoting cardiomyocyte apoptosis. Immunofluorescence analysis revealed a significant increase in reactive oxygen species (ROS) production in LPS-treated cardiomyocytes compared to PBS-treated controls (Figure 2A). Notably, the absence of PKM2 resulted in even higher ROS levels in cardiomyocytes (Figure 2A). Concomitant with the elevated ROS production, we observed a substantial decrease in the activities of antioxidant enzymes such as GSH, GPX, and SOD in response to LPS stimulation (Figure 2B-D). Remarkably, the suppression of antioxidative capacity mediated by LPS was further intensified in *PKM2*-depleted cardiomyocytes (Figure 2B-D). These findings validate that *PKM2* deletion exacerbates LPS-induced oxidative damage in the heart. To assess the pro-apoptotic effects of PKM2 deficiency, we measured caspase-3 activity using an ELISA kit. Following LPS exposure, there was a significant increase in caspase-3 activity, accompanied by a reduction in cell viability as determined by the MTT assay (Figure 2E-F). Intriguingly, in cardiomyocytes lacking PKM2, LPS further enhanced caspase-3 activation and decreased cell viability (Figure 2E-F). These results indicate that *PKM2* deletion amplifies LPS-induced oxidative injury and cell apoptosis in cardiomyocytes.

### *PKM2* Deletion Promotes LPS-Induced Mitochondrial Damage

**Figure 3:**
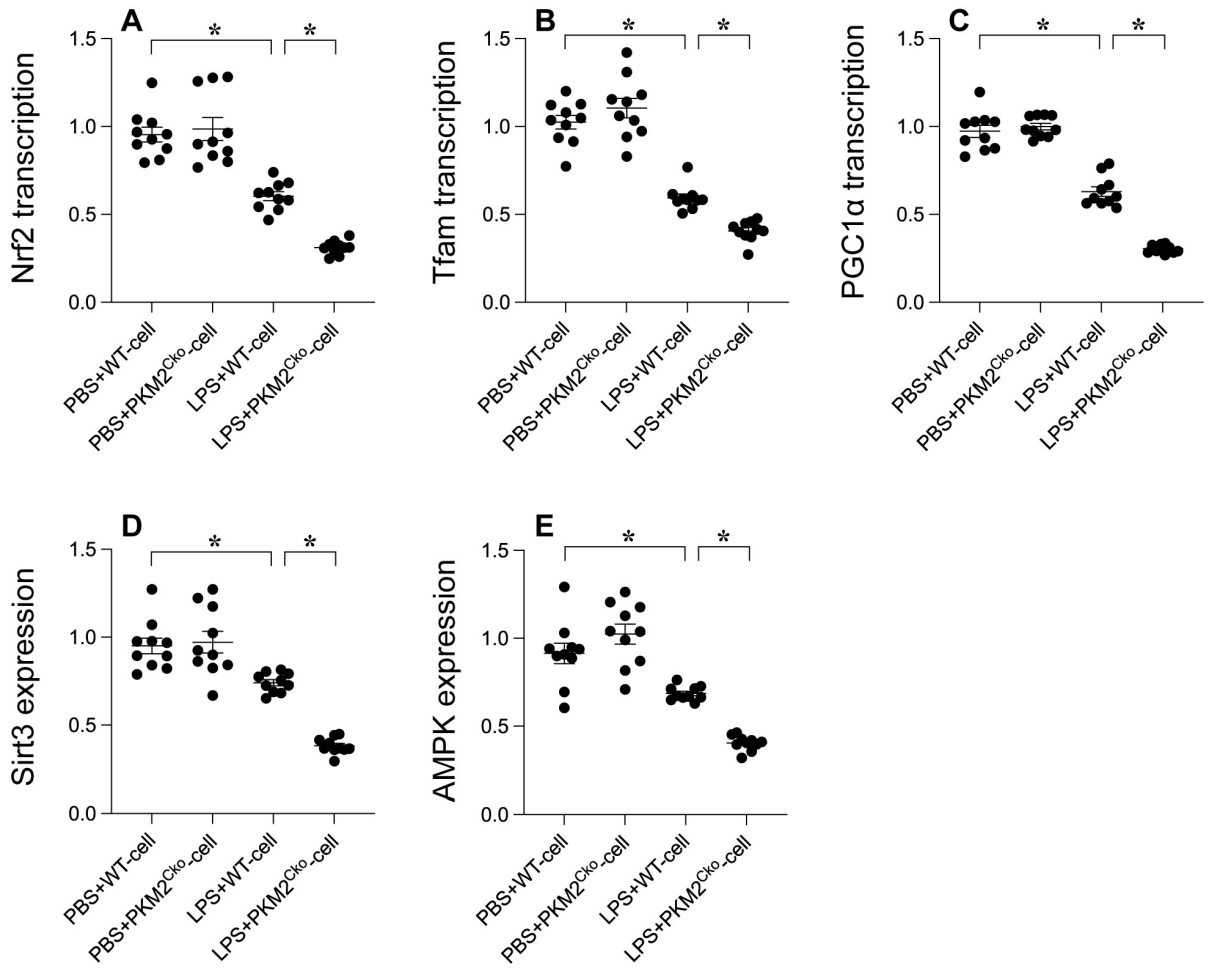
** Influence of *PKM2* Deletion on LPS-Induced Mitochondrial Damag.e A.** Mitochondrial membrane potential was gauged using the JC-1 probe. **B.** ATP levels in cardiomyocytes during LPS exposure were quantified via ELISA.** C-D.** ELISA kits determined mitochondrial respiratory complex I/III activities. *p<0.05.

Excessive oxidative stress can lead to mitochondrial dysfunction. Therefore, we aimed to investigate whether *PKM2* deletion-mediated oxidative stress exacerbates mitochondrial dysfunction in cardiomyocytes. Mitochondrial membrane potential was assessed using immunofluorescence analysis. As depicted in Figure 3A, compared to the control group, LPS treatment reduced mitochondrial membrane potential, a phenomenon that was further heightened in cardiomyocytes isolated from *PKM2* knockout mice (Figure 3A). Given the pivotal role of mitochondria in cardiomyocyte metabolism, we proceeded to evaluate alterations in cardiomyocyte energy metabolism. ATP production was notably decreased in cardiomyocytes exposed to LPS (Figure 3B). Intriguingly, the absence of PKM2 further impeded ATP generation (Figure 3B). Additionally, we observed that the activities of mitochondrial respiratory complexes I and III were downregulated in response to LPS. In PKM2-deficient cardiomyocytes (Figure 3C-D), LPS-induced suppression of mitochondrial respiratory complex activities was further exacerbated. In conclusion, our findings confirm that *PKM2* deletion exacerbates LPS-induced mitochondrial dysfunction in cardiomyocytes.

### *PKM2* Deletion Exacerbates LPS-Mediated Inhibition of Mitochondrial Biogenesis

**Figure 4:**
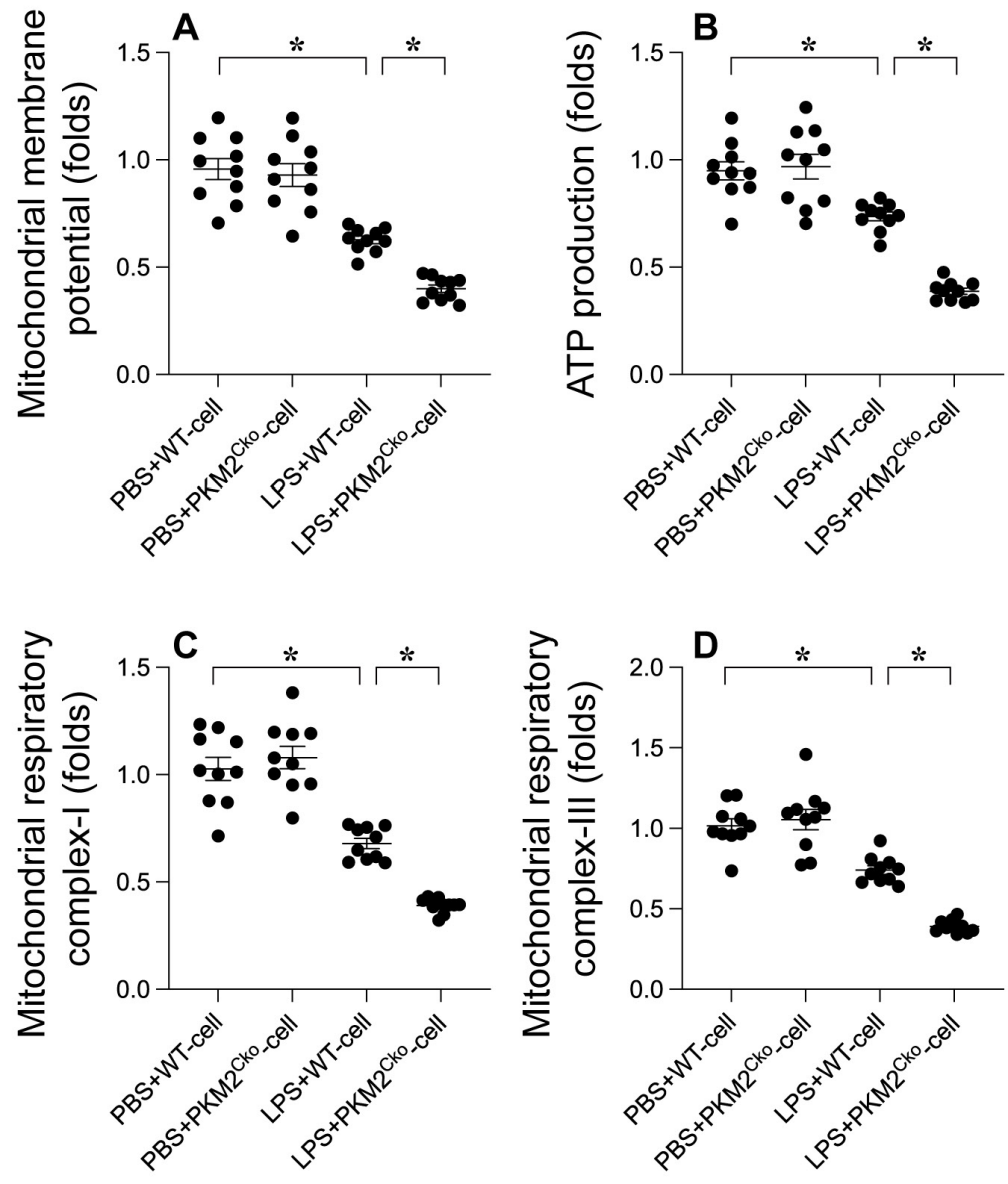
***PKM2* Deletion's Impact on LPS-Mediated Inhibition of Mitochondrial Biogenesis. A-C.** PGC1α, Tfam, and Nrf2 transcription levels were analyzed using qPCR. **D-E.** Sirt3 and AMPK expression in cardiomyocytes under LPS exposure was assessed via Western blotting. *p<0.05.

Mitochondrial biogenesis serves as a protective mechanism against mitochondrial damage during LPS-induced stress. Our goal was to investigate the impact of PKM2 deficiency on mitochondrial integrity through the regulation of mitochondrial biogenesis. Quantitative PCR analysis was conducted to assess key parameters associated with mitochondrial biogenesis, including PGC1α, Tfam, and Nrf2. Following exposure to LPS, the transcription of PGC1α, Tfam, and Nrf2 was significantly reduced (Figure 4A-C), indicating the suppressive effect of LPS on mitochondrial biogenesis. Furthermore, western blot analysis revealed a rapid decrease in the expression of Sirt3 and AMPK in response to LPS exposure (Figure 4D-E). Remarkably, in cardiomyocytes lacking PKM2, the transcription of PGC1α, Tfam, and Nrf2 (Figure 4A-C), as well as the expression of Sirt3 and AMPK (Figure 4D-E), were further inhibited by LPS. Collectively, our findings demonstrate that LPS disrupts mitochondrial biogenesis in septic cardiomyopathy and that this disruption is exacerbated by *PKM2* deletion, highlighting the critical role of PKM2 in the regulation of mitochondrial biogenesis under stress conditions.

### PHB2 Overexpression Mitigates the Delay in Mitochondrial Biogenesis Caused by PKM2 Deficiency

**Figure 5:**
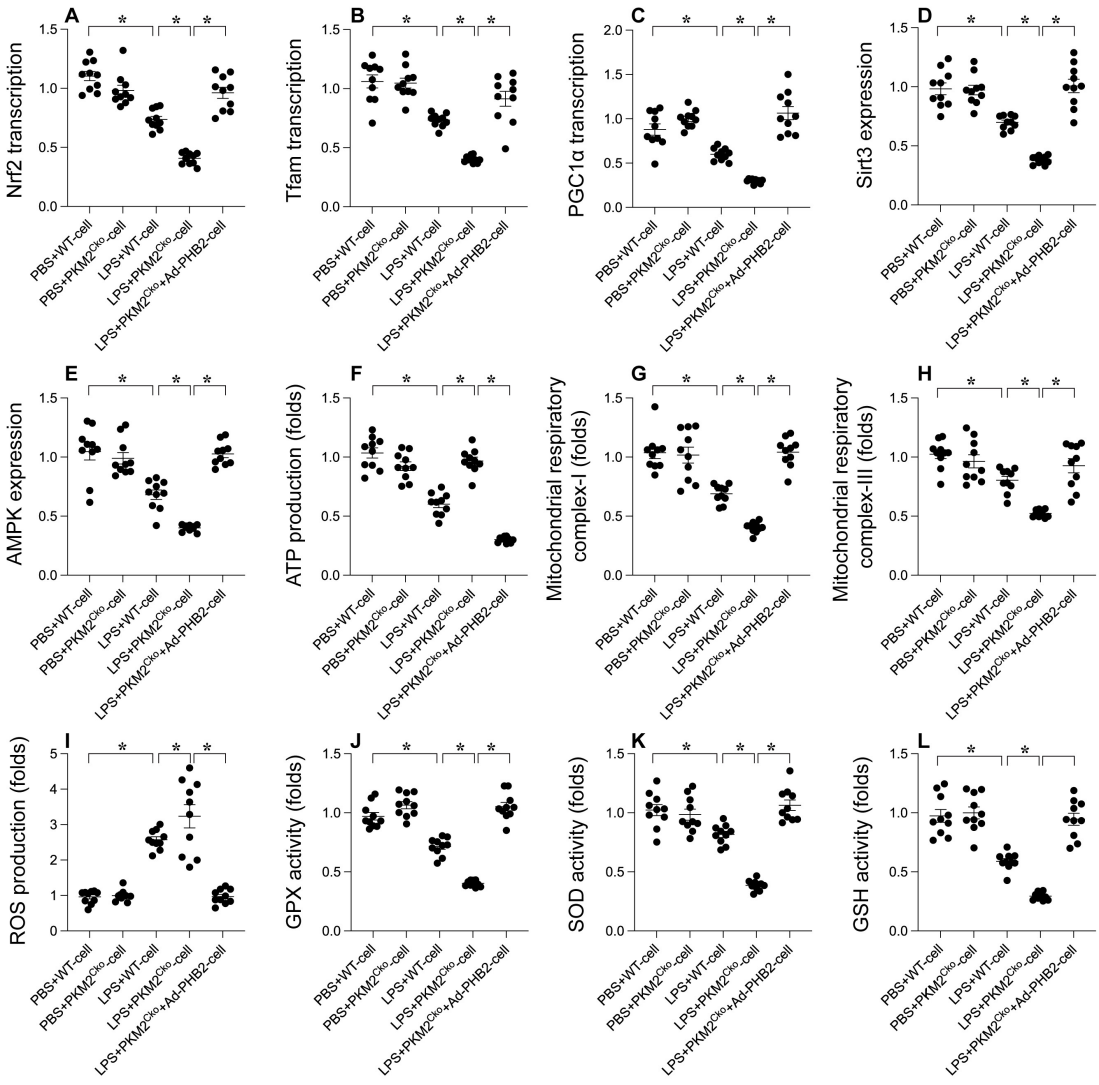
** Alleviating Delay in Mitochondrial Biogenesis with PHB2 Overexpression in PKM2 Deficiency.** Cardiomyocytes were transfected with PHB2 adenovirus (Ad-PHB2) post-LPS treatment to overexpress PHB2. **A-C.** qPCR was used to determine PGC1α, Tfam, and Nrf2 transcription levels. **D-E.** Sirt3 and AMPK expression during LPS exposure were examined via Western blotting. **F.** ATP levels during LPS exposure were measured using an ELISA kit. **G-H.** ELISA kits determined mitochondrial respiratory complex I/III activities. **I.** Immunofluorescence detected ROS levels in cardiomyocytes post-LPS exposure.** J-L.** ELISA kits evaluated GSH, GPX, and SOD activities in response to LPS. *p<0.05.

In order to investigate the potential involvement of PHB2 in the regulatory pathways of PKM2 on mitochondrial biogenesis and in ameliorating LPS-induced cardiomyocyte dysfunction, we conducted experiments involving the overexpression of PHB2 in PKM2-deficient cardiomyocytes. Subsequently, we assessed the impact on mitochondrial biogenesis and function in the presence of LPS. Comparative analysis revealed that, in comparison to *PKM2*-deleted cardiomyocytes, the overexpression of PHB2 reversed the downregulation of key transcription factors involved in mitochondrial biogenesis (Figure 5A-C), namely PGC1α, Tfam, and Nrf2. Furthermore, the expression levels of Sirt3 and AMPK were upregulated following transfection with the PHB2 adenovirus (Figure 5D-E). Additionally, we observed a normalization of mitochondrial function, including ATP production (Figure 5F) and the activities of mitochondrial respiratory complexes I and III (Figure 5G-H), upon PHB2 overexpression when compared to *PKM2*-deleted cardiomyocytes. Moreover, the increase in reactive oxygen species (ROS) production induced by PKM2 deficiency was alleviated by PHB2 overexpression (Figure 5I). Similarly, the disrupted antioxidative capacity of cardiomyocytes resulting from *PKM2* deletion was restored to nearly normal levels upon transfection with the PHB2 adenovirus (Figure 5J-L). These results collectively demonstrate that PHB2 overexpression has the potential to mitigate the inhibition of mitochondrial biogenesis and mitochondrial injury caused by PKM2 deficiency.

## Discussion

Recent research focusing on the involvement of pyruvate kinase M2 (PKM2) in sepsis-induced cardiomyopathy and inflammatory cardiomyopathy has shed light on the intricate molecular mechanisms underlying these conditions. Studies have elucidated the pivotal role of PKM2 in modulating cellular metabolism, energy production, and inflammatory responses within the heart during sepsis and inflammation 14, 15, 38. Interactions of PKM2 with crucial signaling pathways and transcription factors have been highlighted, showcasing its influence on cardiac function and inflammatory processes, thereby impacting the onset and progression of cardiomyopathies. Moreover, recent investigations have delved into the interplay between PKM2 and mitochondrial function 39, 40, revealing significant insights into how PKM2 affects mitochondrial metabolism and cellular energetics in the context of cardiac dysfunction associated with sepsis and inflammation. Promising therapeutic strategies targeting PKM2 and its downstream pathways have shown efficacy in preclinical studies, offering potential avenues for mitigating cardiac damage and enhancing outcomes in septic and inflammatory cardiomyopathies. By unraveling the multifaceted role of PKM2 in these conditions, researchers are paving the way for the development of innovative treatment approaches aimed at restoring metabolic homeostasis, reducing inflammation, and preserving cardiac function in affected individuals. The latest research underscores the critical importance of metabolic regulation and inflammatory signaling in cardiac pathophysiology, providing valuable insights for advancing the management and treatment of these complex cardiovascular conditions. In our current study, knockout experiments were utilized to validate the indispensable role of PKM2 in septic cardiomyopathy. In hearts lacking *PKM2*, significant impairments in contractile function and relaxation were observed, accompanied by exacerbated myocardial inflammation. While our findings align with previous research, further clinical data is warranted to conclusively confirm the essential role of PKM2 in septic cardiomyopathy.

Recent research focusing on the role of prohibitin 2 (PHB2) in sepsis-induced cardiomyopathy and inflammatory cardiomyopathy has yielded significant insights into the molecular mechanisms underlying these conditions. Studies have elucidated the crucial functions of PHB2 in preserving mitochondrial structure, function, and cellular homeostasis within the heart during sepsis and inflammation 41, 42. Interactions of PHB2 with various mitochondrial proteins have been shown to influence mitochondrial dynamics, energy production, and oxidative stress responses, all of which play pivotal roles in the pathogenesis of cardiomyopathies 17, 18, 21, 43. Furthermore, investigations have highlighted the regulatory role of PHB2 in cellular stress responses and apoptosis, indicating its potential in safeguarding cardiac cells from damage in pathological conditions such as sepsis and inflammation 44. The dysregulation of PHB2 in cardiomyopathies has underscored its promise as a therapeutic target for ameliorating mitochondrial dysfunction and enhancing cardiac outcomes in septic and inflammatory scenarios. By targeting pathways associated with PHB2, researchers aim to rejuvenate mitochondrial health, reduce oxidative damage, and improve cardiac function in affected individuals. Overall, recent studies on PHB2 in sepsis-induced cardiomyopathy and inflammatory cardiomyopathy have illuminated the intricate interplay between mitochondrial function, cellular stress responses, and cardiac pathology, providing novel insights into potential treatment strategies for these complex cardiovascular disorders. In our current study, we observed that the overexpression of PHB2 successfully reversed mitochondrial biogenesis activity. Additionally, the abundance of PHB2 was closely linked to mitochondrial integrity and function. This finding offers a fresh perspective on drug development and research targeting mitochondria in the context of septic cardiomyopathy.

Recent research focusing on mitochondrial biogenesis in sepsis-induced cardiomyopathy and inflammatory cardiomyopathy has provided valuable insights into the pathophysiology of these conditions 45, 46. Studies have highlighted the critical role of mitochondrial biogenesis in sustaining cellular energy production and function in the heart during sepsis and inflammation 47, 48. Dysregulation of mitochondrial biogenesis pathways has been associated with mitochondrial dysfunction, compromised energy metabolism, and cardiac damage in these cardiomyopathies 49. Key regulators of mitochondrial biogenesis, such as peroxisome proliferator-activated receptor gamma coactivator 1-alpha (PGC-1α) and nuclear respiratory factors, have been identified, with altered expression observed in septic and inflammatory hearts. Modulation of these factors and their downstream targets has shown promise in preclinical studies as a strategy to restore mitochondrial function and enhance cardiac outcomes in affected individuals 50, 51. Furthermore, studies have underscored the intricate interplay between mitochondrial biogenesis, cellular metabolism, and inflammatory responses in the context of sepsis-induced cardiomyopathy and inflammatory cardiomyopathy. Strategies aimed at boosting mitochondrial biogenesis have been proposed as potential therapeutic interventions to alleviate cardiac dysfunction and inflammation in these conditions. The latest research on mitochondrial biogenesis in sepsis-induced cardiomyopathy and inflammatory cardiomyopathy emphasizes the significance of mitochondrial health and energy metabolism in cardiac function during sepsis and inflammation 52, paving the way for the development of targeted therapies aimed at restoring mitochondrial function and improving outcomes in individuals with these challenging cardiovascular disorders. In our study, we validated the protective effects of mitochondrial biogenesis on mitochondrial integrity. Additionally, our data identified PHB2 as the upstream regulator of mitochondrial biogenesis. The mechanism through which PHB2 modulates mitochondrial biogenesis remains an open question, necessitating further evaluation through animal studies or cellular experiments.

Our study has elucidated the essential role of PKM2 in regulating the progression of septic cardiomyopathy. Deficiency of PKM2 was found to impede mitochondrial biogenesis, leading to a disruption in mitochondrial integrity, ultimately exacerbating myocardial inflammation and heart dysfunction. Interestingly, the overexpression of PHB2 was able to mitigate the detrimental effects of *PKM2* deletion. This novel discovery sheds light on the intricate molecular mechanisms underpinning septic cardiomyopathy, offering a fresh perspective on potential therapeutic targets for this condition.
